# Cardiometabolic Outcomes and Levothyroxine Treatment Effects in Subclinical Hypothyroidism: A Systematic Review

**DOI:** 10.7759/cureus.114468

**Published:** 2026-08-13

**Authors:** Mohamed Abdelgadir Hag Elagib, Marwa A Alamin, Yousif Omer Amin Ahmed, Islam E Musa, Mona A Khaleifa, Elkhansaa A Elsamani, Hiba Najar, Asim Ahmed

**Affiliations:** 1 Internal Medicine, Dallah Namar Hospital, Riyadh, SAU; 2 Internal Medicine, King Fahad Central Hospital, Gizan, SAU; 3 General Medicine, University of Medical Sciences and Technology, Khartoum, SDN; 4 General Practice, Al Quwarah General Hospital, Al Quwarah, SAU; 5 Internal Medicine, Prince Sultan Military Medical City, Riyadh, SAU; 6 Internal Medicine, University Hospital Galway, Galway, IRL; 7 Research, 10 Scholars Academy, Dammam, SAU; 8 Medicine and Surgery, University of Gezira, Wad Madani, SDN

**Keywords:** cardiometabolic risk, dyslipidemia, levothyroxine, subclinical hypothyroidism, systematic review

## Abstract

Subclinical hypothyroidism (SCH) is a common biochemical thyroid disorder characterized by elevated thyroid-stimulating hormone (TSH) concentrations despite normal circulating free thyroxine levels. However, its cardiometabolic consequences and the clinical benefits of levothyroxine therapy remain incompletely established. This systematic review aimed to synthesize clinical evidence on cardiometabolic outcomes in adults with SCH and evaluate the reported effects of levothyroxine therapy or restoration of euthyroidism. Searches were conducted across major biomedical databases and supplementary sources from database inception to August 31, 2025, followed by an updated search through August 4, 2026. Eligible studies included randomized, nonrandomized interventional, and observational studies reporting at least one cardiometabolic outcome. Pooling of clinically comparable studies, including randomized trials reporting lipid outcomes, was considered. However, substantial differences in populations, SCH definitions, comparators, follow-up durations, outcome measures, and the availability of compatible effect estimates and variance data precluded meaningful meta-analysis; findings were synthesized narratively. Fifty reports representing 48 distinct study populations and at least 46,746 participants were included. SCH was associated with adverse lipid, vascular, metabolic, and cardiovascular findings, although the consistency and strength of evidence varied across domains. The strongest available evidence from larger placebo-controlled trials in older adults with mild SCH showed no clinically meaningful improvement in symptoms, carotid atherosclerosis, cardiac function, or overall cardiometabolic biomarkers despite biochemical reductions in TSH. Smaller or selected-population studies reported improvements in some lipid, endothelial, blood pressure, biomarker, or functional outcomes, but these subgroup benefits remain hypothesis-generating rather than established. Overall, the cardiometabolic effects of SCH appear heterogeneous and may vary according to age, disease severity, and comorbid conditions. Current evidence supports individualized, risk-based management rather than routine treatment based on TSH normalization alone.

## Introduction and background

Subclinical hypothyroidism (SCH) is a biochemical thyroid disorder defined by a serum thyroid-stimulating hormone (TSH) concentration above the assay-specific upper reference limit, commonly around 4.0 to 4.5 mIU/L, while circulating free thyroxine (FT4) remains within the laboratory-specific reference range [1-3]. Population-based estimates suggest that SCH affects approximately 4% to 10% of adults, with a higher prevalence among women, older adults, and individuals with thyroid autoimmunity [1-3]. Persistent SCH may indicate early thyroid failure, whereas an isolated elevation in TSH may be transient and may occur in association with aging, low-grade systemic inflammation, or nonthyroidal illness [3].

Current clinical guidance generally distinguishes mild SCH from higher-risk disease, particularly when TSH reaches or exceeds 10 mIU/L [4,5]. This distinction is clinically important because progression to overt hypothyroidism, adverse lipid profiles, and increased cardiovascular risk have been reported more consistently as TSH concentrations become more markedly elevated [4-6]. Thyroid hormone influences lipid metabolism, vascular tone, myocardial performance, body weight, and energy expenditure, providing biological plausibility for cardiometabolic involvement before overt hypothyroidism develops [6].

Epidemiological studies and pooled cohort analyses have linked SCH with coronary heart disease, heart failure, and cardiovascular mortality [7,8]. Associations with coronary events and cardiovascular mortality appear more consistent when TSH concentrations are 10 mIU/L or higher [7-9]. However, the magnitude of these associations varies according to age, baseline cardiovascular risk, specific comorbidities, and the degree of TSH elevation [9]. This variability has contributed to uncertainty about whether SCH should be regarded primarily as a biochemical abnormality or as a potentially modifiable cardiometabolic risk state in selected patients.

Treatment remains controversial. Current guidelines and evidence syntheses do not support routine thyroid hormone therapy for all adults with SCH, particularly when TSH elevation is mild or when patients are older [10-12]. Individualized consideration of levothyroxine therapy may nevertheless be appropriate for selected patients, including younger adults, those with TSH concentrations of 10 mIU/L or higher, and those with dyslipidemia, endothelial dysfunction, hypertension, cardiac disease, or other cardiometabolic risk markers [13]. Existing reviews have often focused on symptoms, quality of life, or broad clinical outcomes, whereas lipid, vascular, cardiac, inflammatory, and metabolic outcomes have not always been examined in sufficient detail. These uncertainties highlight the need for a focused synthesis of cardiometabolic outcomes and treatment-related changes in SCH.

Therefore, this systematic review aimed to synthesize clinical evidence on cardiometabolic outcomes in adults with SCH and to evaluate the reported effects of levothyroxine therapy or restoration of euthyroidism on these outcomes. The review sought to characterize reported cardiometabolic abnormalities and assess treatment-related changes in lipid, vascular, cardiac, inflammatory, and metabolic outcomes. It also compared findings across randomized, nonrandomized interventional, and observational studies; explored variation according to age, sex, baseline TSH level, treatment duration, baseline cardiometabolic risk, diabetes, hypertension, obesity, and established cardiovascular disease; and critically appraised the methodological quality and risk of bias of the included studies.

## Review

Methods

Study Design and Reporting Framework

This systematic review synthesized clinical evidence on cardiometabolic outcomes in adults with SCH and the reported effects of levothyroxine treatment or restoration of euthyroidism. The review was reported in accordance with the Preferred Reporting Items for Systematic Reviews and Meta-analyses 2020 statement (PRISMA 2020), which provides guidance for transparent reporting of study identification, screening, eligibility assessment, and synthesis [14].

The protocol was not registered in PROSPERO. The review questions, clinical eligibility criteria, search concepts, data-extraction fields, synthesis approach, and risk-of-bias assessment plan were specified before the original title and abstract screening. Full-text reports were sought through publisher websites, PubMed Central, institutional or subscription access, and legitimate repositories. Reports that could not be retrieved after reasonable attempts were classified as not retrieved and were not assessed for eligibility. The biochemical eligibility wording reflected the diagnostic approaches used in the included literature. Studies were eligible when elevated TSH was accompanied by thyroid hormone concentrations within the study-specific reference range. FT4-based definitions were preferred, while older studies reporting normal total T4 or total T3 and T4 were retained when the original investigators explicitly classified participants as having SCH, and there was no evidence of overt thyroid dysfunction.

Research Question

This review addressed two related questions. First, which cardiometabolic abnormalities have been reported in adults with SCH? Second, what cardiometabolic outcomes have been reported after levothyroxine treatment, and what outcomes have been associated with restoration of euthyroidism?

Because the review included both exposure-based and intervention-based evidence, observational comparisons of SCH with euthyroid status, levothyroxine treatment studies, and studies reporting outcomes associated with restoration of euthyroidism were interpreted according to their design as distinct evidence streams. The patient, intervention, comparison, and outcome/population, exposure, comparator, outcome, and study design (PICO/PECOS) framework used to define the review question and eligibility structure is shown in Table 1.

**Table 1:** PICO/PECOS framework PICO/PECOS: patient, intervention, comparison, and outcome/population, exposure, comparator, outcome, and study design; BMI: body mass index; FT4: free thyroxine; SCH: subclinical hypothyroidism; T3: triiodothyronine; T4: thyroxine; TSH: thyroid-stimulating hormone PICO/PECOS framework used to define the review question, eligibility criteria, intervention and exposure categories, comparators, outcomes, and eligible study designs

Component	Definition
Population	Adults aged 18 years or older with SCH, defined according to the original study criteria as elevated serum thyroid-stimulating hormone (TSH) with thyroid hormone concentrations within the study-specific reference range. FT4-based definitions were preferred. Older studies reporting normal total T4 or total T3 and T4 were eligible when the original authors explicitly classified participants as having SCH, there was no evidence of overt thyroid dysfunction, and the relevant data were separately extractable. Studies described as mild or borderline hypothyroidism were eligible under the same biochemical conditions
Intervention/exposure	SCH status in observational or controlled comparison studies; levothyroxine treatment; restoration of euthyroidism; or levothyroxine added to standard clinical therapy in relevant cardiometabolic populations
Comparator	Placebo, no treatment, euthyroid controls, non-SCH controls, standard care, or baseline pretreatment values
Outcomes	Lipid profile; blood pressure; glucose metabolism and insulin resistance; BMI or body composition; inflammatory markers; endothelial function; flow-mediated dilation; carotid intima-media thickness or plaque; vascular biomarkers; cardiac function; heart failure outcomes; exercise capacity; cardiovascular events; mortality; and functional status
Study designs	Randomized controlled trials, open-label randomized trials, nonrandomized interventional studies, before-and-after studies, prospective or retrospective cohort studies, case-control studies, cross-sectional studies, and controlled clinical studies

Eligibility Criteria

The inclusion and exclusion criteria applied during study selection are summarized in Table 2.

**Table 2:** Eligibility criteria FT4: free thyroxine; SCH: subclinical hypothyroidism; T3: triiodothyronine; T4: thyroxine; TSH: thyroid-stimulating hormone Predefined population, biochemical, intervention or exposure, outcome, study design, report, and full-text criteria used for study selection

Domain	Inclusion criteria	Exclusion criteria
Population	Human clinical studies involving adults aged 18 years or older. Mixed-age studies were eligible only when adult data were separately extractable	Pediatric-only studies, pregnancy-only studies, or mixed-age studies without separately extractable adult data
SCH definition	Elevated TSH with thyroid hormone concentrations within the study-specific reference range. FT4-based definitions were preferred. Older studies reporting normal total T4 or total T3 and T4 were accepted when the original authors explicitly classified participants as having SCH, and there was no evidence of overt thyroid dysfunction. Mild or borderline labels were accepted only when the reported biochemistry supported SCH	Overt hypothyroidism, hyperthyroidism, thyroid hormone concentrations outside the applicable reference range, thyroid dysfunction without sufficient biochemical information to verify SCH, or mixed thyroid groups without separately extractable SCH data
Exposure/intervention	SCH status, levothyroxine treatment, restoration of euthyroidism, or levothyroxine added to standard therapy	Studies not evaluating SCH or an eligible levothyroxine-related exposure or intervention
Outcomes	At least one extractable cardiometabolic outcome within the prespecified lipid, vascular, cardiac, metabolic, inflammatory, or clinical cardiovascular domains	No eligible cardiometabolic outcome or insufficient outcome data for extraction
Study design	Randomized, nonrandomized interventional, controlled clinical, before-and-after, cohort, case-control, or cross-sectional studies	Case reports, editorials, letters, narrative reviews, systematic reviews, meta-analyses, protocols without results, animal studies, or in vitro studies
Report characteristics	Original research published in English. For mixed human and animal studies, only separately extractable human data were eligible	Nonoriginal publications, non-English reports, or mixed human and animal studies without separately extractable human data
Full-text availability	Full text lawfully available to the review team through publisher pages, PubMed Central, institutional or subscription access, or legitimate repositories	Reports that could not be retrieved after reasonable attempts were classified as not retrieved and were not assessed for eligibility; they were not counted as full-text exclusions

Statistical significance was not an eligibility criterion, and studies reporting null findings were retained when all other criteria were met. Eligibility was based on the full report rather than the abstract alone.

Information Sources and Search Strategy

The original comprehensive database search was conducted from inception to August 31, 2025, in PubMed/MEDLINE, Embase, Scopus, Web of Science, and the Cochrane Central Register of Controlled Trials (CENTRAL). Google Scholar was used as a supplementary source, and the reference lists of included studies and relevant reviews were screened manually to identify additional eligible reports.

Before resubmission, a targeted update search was conducted on August 4, 2026, covering September 1, 2025, through August 4, 2026. PubMed/MEDLINE was searched using the original core concepts with the update date limits. This was supplemented by the Scopus Research Question tool using the natural-language question: “In adults with subclinical hypothyroidism, what cardiometabolic abnormalities have been reported, and what effects do levothyroxine treatment or restoration of euthyroidism have on these outcomes?” The Scopus tool was used only as a supplementary discovery source; all eligibility decisions were made manually by the reviewers. Records outside the predefined update period were removed before screening.

Searches were restricted to English-language publications. The strategies combined terms for SCH, thyroid dysfunction, cardiometabolic and cardiovascular outcomes, lipid profile, blood pressure, insulin resistance, glucose metabolism, inflammation, endothelial and vascular outcomes, cardiac function, heart failure, and levothyroxine therapy. For Google Scholar, the first 100 relevance-ranked results were screened. The database- and source-specific strategies and final search dates are presented in Table 3.

**Table 3:** Search strategies and final search dates CENTRAL: Cochrane Central Register of Controlled Trials; CRP: C-reactive protein; IMT: intima-media thickness; SCH: subclinical hypothyroidism Database- and source-specific search strategies used to identify studies of SCH, levothyroxine treatment, and cardiometabolic outcomes. The original searches in Embase, Scopus, Web of Science, CENTRAL, and Google Scholar were completed on August 31, 2025; the PubMed/MEDLINE and supplementary Scopus update was completed on August 4, 2026

Source and final search date	Search strategy
PubMed/MEDLINE final search: August 4, 2026	("subclinical hypothyroidism" OR "subclinical thyroid dysfunction" OR "mild hypothyroidism" OR "borderline hypothyroidism") AND (cardiometabolic OR cardiovascular OR cholesterol OR lipid OR LDL OR HDL OR triglycerides OR dyslipidemia OR "blood pressure" OR hypertension OR "insulin resistance" OR glucose OR "metabolic syndrome" OR inflammation OR CRP OR endothelial OR "flow-mediated dilation" OR "intima-media thickness" OR "carotid IMT" OR cardiac OR "heart failure" OR echocardiography OR "exercise capacity" OR levothyroxine OR "L-thyroxine" OR thyroxine OR "thyroid hormone replacement"). The update applied a Create Date limit of September 1, 2025, to August 4, 2026
Embase final search: August 31, 2025	('subclinical hypothyroidism' OR 'subclinical thyroid dysfunction' OR 'mild hypothyroidism' OR 'borderline hypothyroidism') AND (cardiometabolic OR cardiovascular OR lipid OR cholesterol OR dyslipidemia OR 'blood pressure' OR hypertension OR 'insulin resistance' OR glucose OR 'metabolic syndrome' OR inflammation OR endothelial OR 'flow mediated dilation' OR 'intima media thickness' OR 'carotid intima media thickness' OR cardiac OR 'heart failure' OR echocardiography OR 'exercise capacity' OR levothyroxine OR 'l-thyroxine' OR thyroxine OR 'thyroid hormone replacement')
Scopus (original advanced search) final search: August 31, 2025	TITLE-ABS-KEY(("subclinical hypothyroidism" OR "subclinical thyroid dysfunction" OR "mild hypothyroidism" OR "borderline hypothyroidism") AND (cardiometabolic OR cardiovascular OR cholesterol OR lipid OR dyslipidemia OR "blood pressure" OR hypertension OR "insulin resistance" OR glucose OR "metabolic syndrome" OR inflammation OR endothelial OR "flow-mediated dilation" OR "intima-media thickness" OR "carotid IMT" OR cardiac OR "heart failure" OR echocardiography OR "exercise capacity" OR levothyroxine OR "L-thyroxine" OR thyroxine OR "thyroid hormone replacement"))
Scopus research question (supplementary update) final search: August 4, 2026	Natural-language question: “In adults with subclinical hypothyroidism, what cardiometabolic abnormalities have been reported, and what effects do levothyroxine treatment or restoration of euthyroidism have on these outcomes?” Records within the predefined update period were cross-checked against PubMed/MEDLINE.
Web of Science final search: August 31, 2025	TS=(("subclinical hypothyroidism" OR "subclinical thyroid dysfunction" OR "mild hypothyroidism" OR "borderline hypothyroidism") AND (cardiometabolic OR cardiovascular OR cholesterol OR lipid OR dyslipidemia OR "blood pressure" OR hypertension OR "insulin resistance" OR glucose OR "metabolic syndrome" OR inflammation OR endothelial OR "flow-mediated dilation" OR "intima-media thickness" OR "carotid IMT" OR cardiac OR "heart failure" OR echocardiography OR "exercise capacity" OR levothyroxine OR "L-thyroxine" OR thyroxine OR "thyroid hormone replacement"))
Cochrane CENTRAL final search: August 31, 2025	("subclinical hypothyroidism" OR "subclinical thyroid dysfunction" OR "mild hypothyroidism") AND (levothyroxine OR thyroxine OR cardiovascular OR cardiometabolic OR lipid OR cholesterol OR endothelial OR carotid OR cardiac OR "heart failure" OR insulin resistance OR blood pressure)
Google Scholar final search: August 31, 2025	"subclinical hypothyroidism" cardiometabolic levothyroxine cholesterol LDL triglycerides endothelial "carotid IMT" insulin resistance "heart failure"; first 100 relevance-ranked results screened

Record Management and Deduplication

Records from the original database searches were imported into Zotero for reference management, and duplicates were identified using Zotero’s duplicate-detection function before screening. The original library and the updated PubMed/MEDLINE and Scopus outputs were managed in MS Excel (Microsoft Corporation, Redmond, Washington, United States) for screening documentation and updated-search integration. Excel checks were used to confirm duplicates identified by Zotero and to identify any additional duplicates that Zotero may have missed, using article title, author names, publication year, journal name, and DOI when available. The supplementary Scopus output was cross-checked against the PubMed/MEDLINE update by DOI and normalized title to prevent double-counting.

Study Selection

Mohamed Abdelgadir Hag Elagib (MAHE) and Marwa A. Alamin (MAA) independently screened titles and abstracts against the eligibility criteria described above. Records were excluded at this stage when they involved an ineligible population, did not evaluate SCH or an eligible levothyroxine-related exposure or intervention, did not report a relevant cardiometabolic outcome, or represented an ineligible publication type or study design. Full texts of potentially eligible reports were then sought and independently assessed by the same reviewers.

Studies described as mild, borderline, or SCH were included when the reported biochemical thresholds or baseline laboratory values confirmed elevated TSH with thyroid hormone concentrations within the study-specific reference range and the relevant data were separately extractable. FT4-based definitions were preferred, while older studies reporting normal total T4 or total T3 and T4 were retained when the original authors explicitly classified participants as having SCH, and there was no evidence of overt thyroid dysfunction. Reports that could not be obtained after searches of publisher pages, PubMed Central, available institutional or subscription access, and legitimate repositories were classified as not retrieved. Disagreements were resolved through discussion and consensus. Reasons for exclusion were recorded at full-text assessment, and the selection process was documented in a PRISMA 2020 flow diagram [14].

Handling of Multiple or Overlapping Reports

Potentially overlapping reports were identified by comparing author groups, institutions and study settings, recruitment periods, eligibility criteria, sample sizes, baseline participant characteristics, interventions or exposures, comparators, outcome-assessment schedules, and follow-up periods. When overlap was not explicitly reported, these features were used to link publications. Reports from the same or related cohorts were retained as separate publications only when they contributed distinct outcomes, follow-up periods, or substudy questions. Such reports were treated as companion or secondary reports and were not counted as additional independent studies.

Data Extraction

Data extraction was performed independently by MAHE and MAA using a structured Microsoft Excel form. Extracted variables included first author, publication year, country, journal, study design, participant characteristics, SCH definition, sample size, intervention or exposure, comparator, treatment duration, follow-up duration, cardiometabolic outcomes, direction and magnitude of effect, statistical significance, and the main study conclusion.

Where available, extracted outcome data included baseline and follow-up values, between-group differences, measures of variance, 95% confidence intervals, p-values, and units of measurement. For interventional studies, between-group treatment effects were prioritized over within-group changes when available. For observational studies, adjusted estimates were prioritized over unadjusted estimates. The two reviewers cross-checked extracted data for completeness and accuracy, and inconsistencies were resolved through discussion and consensus. Missing or nonextractable numerical data were not imputed.

Outcome Domains

The cardiometabolic outcome domains were prespecified before study screening based on the review questions, eligibility criteria, and structured extraction form. Lipid outcomes included total cholesterol, low-density lipoprotein cholesterol (LDL-C), high-density lipoprotein cholesterol (HDL-C), triglycerides, and dyslipidemia prevalence. Vascular outcomes included endothelial function, flow-mediated dilation, carotid intima-media thickness (CIMT), carotid plaque, and vascular biomarkers. Cardiac and heart failure outcomes included echocardiographic measures, left ventricular ejection fraction, diastolic function, N-terminal pro-B-type natriuretic peptide, six-minute walk distance, New York Heart Association functional class, atrial fibrillation, heart failure hospitalization, cardiovascular death, mortality, and composite cardiovascular events.

Metabolic outcomes included body mass index (BMI), body composition, fasting or postprandial glucose, glycated hemoglobin, insulin-resistance indices, homeostatic model assessment of insulin resistance (HOMA-IR), triglyceride-glucose index, and metabolic syndrome-related markers. Inflammatory and vascular biomarker outcomes included C-reactive protein, high-sensitivity C-reactive protein, homocysteine, oxidized LDL, endocan, endothelin-1, asymmetric dimethylarginine, apelin, and related markers.

Risk-of-Bias Assessment

Risk of bias was assessed using design-appropriate tools for the cardiometabolic outcomes most relevant to this review. Randomized controlled trials and randomized open-label trials were assessed using the Cochrane Risk of Bias 2 tool (RoB 2), which evaluates bias arising from the randomization process, deviations from intended interventions, missing outcome data, measurement of the outcome, and selection of the reported result [15]. An overall judgment of low risk required low risk in all domains; some concerns indicated at least one domain with some concerns and no high-risk domain; and high risk indicated at least one high-risk domain or multiple concerns that substantially reduced confidence in the result.

Nonrandomized interventional studies, controlled treatment-response studies, and before-and-after studies were assessed using Risk of Bias in Nonrandomized Studies of Interventions​​​ (ROBINS-I) [16]. The domains were confounding, selection of participants, classification of interventions, deviations from intended interventions, missing data, outcome measurement, and selection of the reported result. Judgments were low, moderate, serious, or critical risk of bias. Potential confounding domains were identified according to the study population and outcome and included age, sex, baseline TSH concentration, BMI or obesity, diabetes, hypertension, established cardiovascular disease, baseline lipid profile, other relevant comorbidities, and concomitant cardiometabolic treatment. For uncontrolled before-and-after studies, assessment focused on bias in the estimated pre-post intervention change, with particular attention to confounding, cointerventions, time-related changes, regression to the mean, participant selection, missing data, outcome measurement, and selective reporting. The absence of a concurrent control group was reflected in the confounding and overall judgments.

Observational exposure studies, including cross-sectional, retrospective cohort, prospective cohort, and case-control studies, were assessed using a predefined observational quality checklist. The checklist evaluated selection and sampling, comparability and confounding, exposure definition, outcome measurement, statistical analysis, and overall interpretability. Confounding was judged using the same population- and outcome-specific factors described above. Overall judgments were based on the importance of limitations across domains rather than a numerical score and were categorized as low concern, some concerns, moderate concerns, or serious concerns.

Risk-of-bias assessment was conducted independently by MAHE and MAA. Inter-reviewer agreement was not formally quantified. Differences in judgment were resolved through discussion and consensus, and domain-level judgments informed the overall interpretation of the evidence.

Statistical Analysis and Data Synthesis

Before selecting narrative synthesis, the reviewers examined whether clinically comparable studies could be pooled, particularly randomized levothyroxine trials reporting total cholesterol or LDL-C. Meaningful meta-analysis was not considered appropriate because studies differed substantially in design, intervention or exposure, comparator, participant age and baseline risk, SCH definition and TSH concentration, treatment and follow-up duration, outcome definition and measurement, units, and statistical reporting. Several studies also lacked compatible between-group effect estimates or variance data. Effect estimates and 95% confidence intervals were reported when available from the primary studies, followed by p-values where relevant. No new effect estimates, confidence intervals, or p-values were calculated, and missing values were not imputed.

Findings were therefore synthesized narratively by outcome domain and by intervention- or exposure-based evidence stream. The synthesis was organized into lipid outcomes; endothelial and vascular outcomes; cardiac function, heart failure, and cardiovascular events; insulin resistance and broader metabolic outcomes; inflammatory and vascular biomarkers; and subgroup-specific findings. Within each domain, studies were summarized according to design, intervention or exposure, comparator, direction and magnitude of effect, statistical significance, and risk-of-bias judgment.

Randomized placebo-controlled levothyroxine trials were interpreted as the strongest evidence for treatment effects. Open-label randomized and nonrandomized treatment studies were interpreted as supportive clinical evidence, while observational studies were interpreted as evidence of association rather than treatment effect. Where possible, findings were considered according to age, sex, baseline TSH level, TSH below 10 mIU/L versus 10 mIU/L or higher, baseline cardiometabolic risk, obesity, hypertension, diabetes, established cardiovascular disease, heart failure phenotype, and treatment duration. Potential publication bias was considered qualitatively, particularly when positive findings came from small, uncontrolled, or before-and-after studies. Formal assessment of publication bias was not performed because meta-analysis was not undertaken and clinically comparable effect estimates were insufficient for funnel-plot or regression-based assessment.

Certainty of Evidence

Formal certainty-of-evidence grading was not performed. Conclusions were therefore based on consistency of findings, study design, risk of bias, directness and clinical relevance of outcomes, sample size, and the distinction between randomized levothyroxine evidence, supportive nonrandomized treatment evidence, and observational association evidence. The absence of formal certainty-of-evidence grading was acknowledged as a limitation.

Results

Study Selection

The original search identified 641 records. After removal of 120 duplicate records and 21 records marked as ineligible before screening, 500 records were screened by title and abstract. Of these, 135 records were excluded, and 365 full-text reports were assessed. After full-text review, 340 reports were excluded because of an irrelevant exposure, intervention, or clinical focus (n = 120), an ineligible study design (n = 70), a wrong population (n = 62), an ineligible outcome (n = 40), conference-abstract-only publication (n = 30), or an overlapping dataset (n = 18). Twenty-five reports were included in the original synthesis. Three related reports arose from the Thyroid Hormone Replacement for Untreated Older Adults With Subclinical Hypothyroidism (TRUST) population; therefore, the original 25 reports represented 23 distinct study populations.

The update search retrieved 289 records: 189 from PubMed/MEDLINE and 100 from the supplementary Scopus research question export. Before screening, 96 Scopus records outside the predefined update period and three cross-source duplicates were removed, leaving 190 records. Of these, 152 were excluded during title and abstract screening, and 38 reports were sought for retrieval. Seven reports could not be retrieved. Thirty-one full-text reports were assessed, and six were excluded because the biochemical SCH criteria were not met (n = 3) or SCH-specific cardiometabolic outcomes were not separately extractable (n = 3). Twenty-five reports were included from the update.

**Figure 1:**
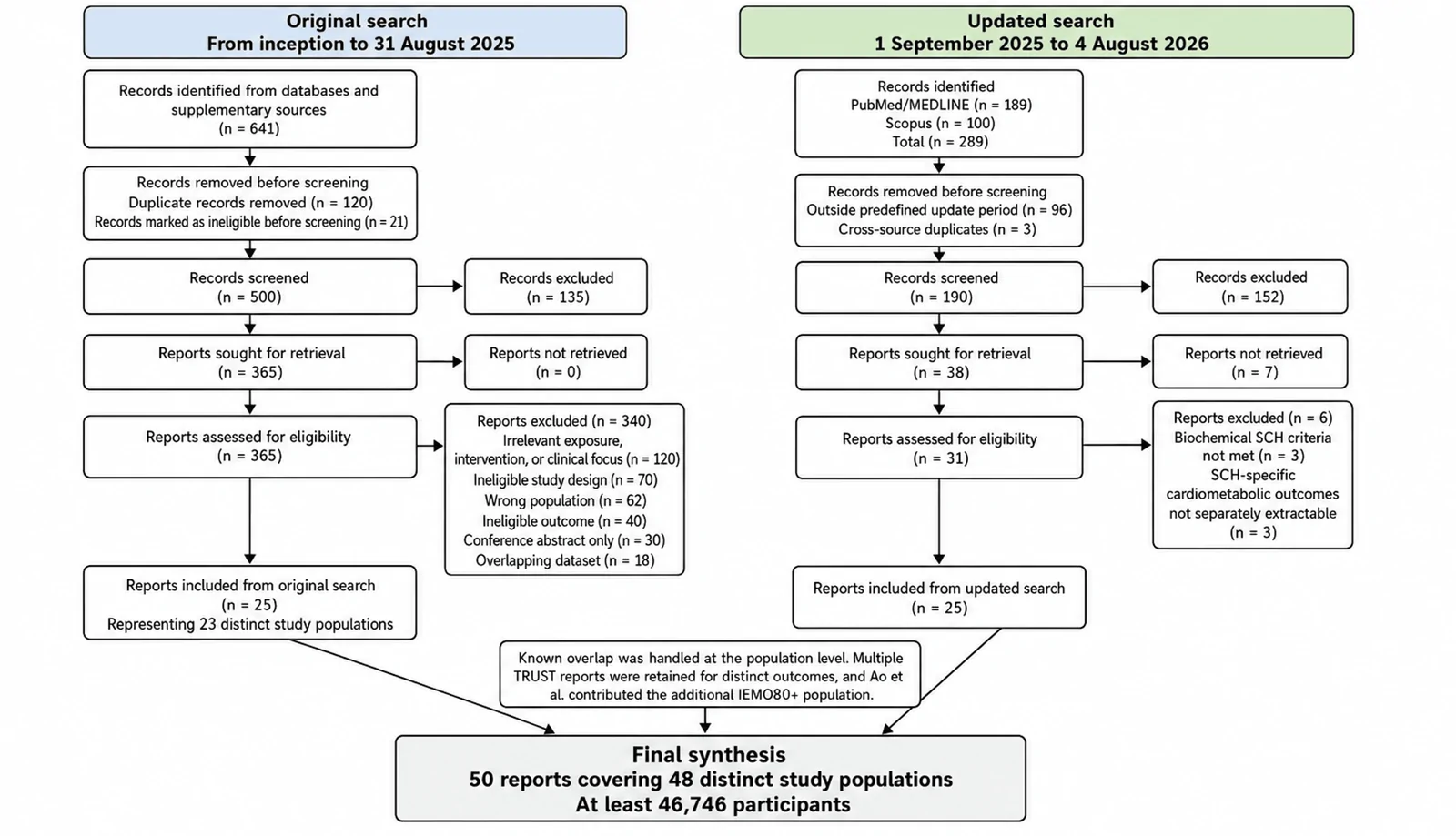
PRISMA 2020 flow diagram of study identification, screening, eligibility assessment, and inclusion PRISMA: Preferred Reporting Items for Systematic Reviews and Meta-analyses; IEMO80+: Institute for Evidence-Based Medicine in Old Age 80-plus thyroid trial; SCH: subclinical hypothyroidism; TRUST: Thyroid Hormone Replacement for Untreated Older Adults With Subclinical Hypothyroidism The original search, conducted from database inception to August 31, 2025, yielded 25 eligible reports representing 23 distinct study populations. The updated search, covering September 1, 2025, to August 4, 2026, identified 25 additional eligible reports. Multiple publications from the TRUST trial were retained because they reported distinct outcomes, while the pooled report by Ao et al. included participants from both TRUST and the additional IEMO80+ population [52]. After accounting for overlapping reports at the population level, the final synthesis included 50 reports representing 48 distinct study populations and at least 46,746 participants

One report identified during the update pooled data from TRUST and IEMO80+. TRUST participants overlapped with reports already included, whereas IEMO80+ contributed an additional trial population. After accounting for all related reports at the population level, the final synthesis comprised 50 reports covering 48 distinct study populations. The selection process is summarized in Figure 1.

Study Characteristics

The final synthesis comprised 50 reports covering 48 distinct study populations. Based on the analyzable sample sizes reported in the included publications, these populations comprised at least 46,746 participants. This conservative minimum avoided double-counting known overlapping reports and used 8,000 as the lower bound for the study described as including more than 8,000 adults. To avoid overlapping design labels, reports were grouped by the primary evidence stream used in this review: randomized studies (n = 8 reports), nonrandomized interventional studies (n = 14), and observational studies (n = 28). Reports containing secondary cross-sectional or longitudinal components were interpreted according to the limitations of those components but were counted once under their primary evidence stream. The evidence included older adults, women with polycystic ovary syndrome (PCOS) or menopause-related cohorts, people with obesity or diabetes, hospitalized patients, and patients with heart failure or coronary artery bypass grafting. Outcomes covered lipid and glucose metabolism, insulin resistance, inflammatory and vascular biomarkers, endothelial and structural vascular measures, cardiac function, arrhythmia, cardiovascular events, mortality, and functional status. The characteristics of the included reports are summarized in Table 4.

**Table 4:** Characteristics of the included reports ADMA: asymmetric dimethylarginine; AF: atrial fibrillation; AIP: atherogenic index of plasma; ApoA1/ApoB: apolipoproteins A1/B; BMI: body mass index; BP: blood pressure; CABG: coronary artery bypass grafting; CIMT/IMT: carotid/intima-media thickness; CRP/hs-CRP: C-reactive protein/high-sensitivity C-reactive protein; E/e′: early mitral inflow to annular velocity ratio; ECG: electrocardiogram; ET-1: endothelin-1; FMD: flow-mediated dilation; FT3/FT4: free triiodothyronine/free thyroxine; HbA1c: glycated hemoglobin; HDL-C/LDL-C: high-/low-density lipoprotein cholesterol; HFmrEF/HFpEF/HFrEF: heart failure with mildly reduced/preserved/reduced ejection fraction; HOMA-IR: homeostatic model assessment of insulin resistance; ICAM-1: intercellular adhesion molecule 1; Lp(a): lipoprotein(a); LVEF: left ventricular ejection fraction; MACE: major adverse cardiovascular events; NT-proBNP: N-terminal pro-B-type natriuretic peptide; NYHA: New York Heart Association; ox-LDL: oxidized low-density lipoprotein; PCOS: polycystic ovary syndrome; RCT: randomized controlled trial; SCH: subclinical hypothyroidism; T2DM: type 2 diabetes mellitus; TSH/TSHR: thyroid-stimulating hormone/receptor; TyG: triglyceride-glucose index; NHANES: National Health and Nutrition Examination Survey Characteristics of 50 reports covering 48 distinct study populations. Reports were classified by the primary evidence stream used in the synthesis

Study ID	Country/design	Population and sample size	SCH definition	Intervention/exposure	Comparator	Follow-up	Main outcomes
Niknam et al. [17]	Iran; quasi-experimental	SCH patients and controls; total n = 50	TSH >4 mIU/L, normal FT4	Levothyroxine 50 µg/day	Controls and baseline	2 months	FMD, IMT, TSH, lipids
Shahebrahimi et al. [18]	Iran; double-blind RCT	SCH patients; total n = 100	TSH >4.70 µIU/mL, normal FT4	Levothyroxine 50 to 75 µg	Placebo	3 to 6 months	TC, LDL, HDL, TG, lipoprotein(a)
Beysel et al. [19]	Turkey; retrospective before-after	SCH patients and controls; total n = 70	TSH >4.0 mIU/mL, normal FT3/FT4	Levothyroxine 50 µg/day	Controls and baseline	3 months	Lipids, BMI, BP, hs-CRP, glucose
Adamarczuk-Janczyszyn et al. [20]	Poland; before-after interventional	Obese postmenopausal women; total n = 187	TSH >4.0 µIU/mL, normal FT4	L-thyroxine therapy	Controls and baseline	6 months	Lipids, BP, BMI, CRP, homocysteine
Evran et al. [21]	Turkey; open-label randomized trial	Drug-naive SCH patients; total n = 53	Elevated TSH, normal FT3/FT4	Levothyroxine replacement	No treatment	6 months	Echocardiography, lipids, symptoms, BP
Orel and Martynyuk [22]	Ukraine; before-after trial	SCH with hypertension; total n = 31	Elevated TSH, normal FT4	Levothyroxine plus antihypertensive therapy	Baseline	3 months	Lipids, FMD, cognition
Stott et al. [23]	Europe; double-blind RCT	Adults ≥65 years; total n = 737	TSH 4.60 to 19.99 mIU/L, normal FT4	Levothyroxine adjusted by TSH	Placebo	1 to 3 years	Symptoms, tiredness, quality of life, BP, BMI
Blum et al. [24]	Switzerland; double-blind RCT	Adults ≥65 years; total n = 185	TSH 4.60 to 19.99 mIU/L, normal FT4	Levothyroxine titrated to TSH normalization	Placebo	Median 18.4 months	CIMT, carotid plaque, plaque thickness
Gencer et al. [25]	Switzerland; double-blind RCT	Adults ≥65 years; total n = 185	TSH 4.60 to 19.99 mIU/L, normal FT4	Levothyroxine titrated to TSH normalization	Placebo	Median 18.4 months	LVEF, E/e′, NT-proBNP, hs-CRP, lipids
Yasar et al. [26]	Turkey; prospective case-control with reassessment	SCH due to Hashimoto’s thyroiditis and controls; total n = 246	Elevated TSH, normal FT4	Levothyroxine until euthyroid state	Controls and baseline	12 weeks	Irisin, apelin, homocysteine, CIMT, HOMA-IR, lipids
Gao et al. [27]	China; cross-sectional survey	Women undergoing thyroid and lipid testing; total n = 1,397	Elevated TSH consistent with SCH	SCH status	Normal thyroid function	Not applicable	TSH, TG, lipid abnormalities
Cabral et al. [28]	Brazil; randomized open-label prospective trial	Female mild SCH patients; total n = 32	TSH 4.0 to 12.0 mIU/mL, normal FT4	L-thyroxine replacement	No treatment	12 months	FMD, CIMT, lipids
Pandrc et al. [29]	Serbia; prospective before-after trial	Untreated SCH patients; total n = 35	TSH above normal to 10 mIU/L, normal FT4	Levothyroxine 25 to 75 µg	Baseline	3 months after euthyroidism	Lipids, BP, BMI, HOMA-IR, homocysteine, quality of life
Pala et al. [30]	India; prospective matched case-control	SCH patients and controls; total n = 500	TSH ≤10 or >10 mIU/L, normal total T4	Levothyroxine in TSH >10 group	Euthyroid controls	3 months	Lipids, TSH, thyroid hormones
Maurya et al. [31]	India; prospective nonrandomized interventional study	SCH patients and controls; total n = 44	Raised TSH, normal total T3/T4	Levothyroxine 25 to 75 µg	Healthy controls and baseline	3 months	Lipids, TSH, T3, T4
Goyal et al. [32]	India; retrospective cross-sectional	Perimenopausal females; total n = 100	Elevated TSH, normal FT3/FT4	SCH status	Euthyroid females	Not applicable	Lipids, atherogenic indices
Ejaz et al. [33]	Pakistan; longitudinal/case-control	Adults aged 40 to 70 years; total n = 900	TSH 5 to 10 mIU/L, normal FT3/FT4	SCH status	Non-SCH participants	Not specified	Lipids, BMI, hypertension, diabetes
Kirac et al. [34]	Turkey; retrospective study	Obese women; total n = 78	Elevated TSH, normal FT4	SCH status	Euthyroid obese women	Not applicable	HOMA-IR, TyG index, TG, glucose
Tan et al. [35]	China; prospective observational cohort	HFpEF patients; completed follow-up n = 492	TSH >4.49 mIU/L, normal FT4	SCH status in HFpEF	Euthyroid HFpEF	Mean 33 months	Cardiovascular events, rehospitalization, cardiovascular death, FMD
Harrar et al. [36]	Morocco; retrospective cross-sectional	Hospital patients; total n = 447	TSH >4.0 µIU/mL, normal FT4	SCH status	Euthyroid individuals	Not applicable	Lipids, TSH, FT4
Alzahrani et al. [37]	Saudi Arabia; retrospective cohort	Adults matched by age, sex, BMI; total n = 208	TSH 4.5-10 mIU/L, normal total T4	Levothyroxine treatment	Controls and baseline	Retrospective 2016 to 2022	Lipids, vitamin D, HbA1c, glucose, BP
Zhu and Jiang [38]	China; retrospective community study	Community residents; total n = 2,119	Elevated TSH, normal FT3/FT4	SCH status	Non-SCH group	Not applicable	Lipids, glucose, BMI, BP
Hewage et al. [39]	Sri Lanka; cross-sectional	Nondiabetic women aged 18 to 35; total n = 282	TSH 4.94 to 10 mIU/mL, normal FT4/T3	SCH status	Euthyroid women	Not applicable	HOMA-IR, lipids, obesity indices, BP
Serdar Hiršl et al. [40]	Croatia; prospective case-control	Mild SCH and controls; total n = 91	TSH 4.2 to 10.0 mIU/L, normal FT4	Levothyroxine	Controls and baseline	6 months after euthyroidism	Endocan, ADMA, ET-1, lipids, hs-CRP
Wang et al. [41]	China; multicenter open-label RCT	SCH with HFrEF; total n = 117	TSH above normal to 10 mIU/L, normal FT4	Low-dose levothyroxine plus HFrEF therapy	Standard HFrEF therapy	24 weeks	Six-minute walk distance, NYHA, TSH, NT-proBNP, Minnesota Living with Heart Failure Questionnaire
Rahm et al. [42]	Germany; retrospective clinical cohort with experimental component	Adults with SCH and ECG within 7 days; n = 2,311	Elevated TSH with normal FT3/FT4; TSH 4-10 vs >10 mIU/L	SCH severity/TSH level; human component only	Lower-TSH SCH group	Cross-sectional clinical assessment	AF prevalence, ECG parameters, cardiac electrophysiology
Tsilingiris et al. [43]	Greece; matched cross-sectional study with longitudinal treatment subset	SCH n = 120 and euthyroid controls n = 120; treated severe SCH subset n = 16	TSH ≥4 mIU/L on two measurements with thyroid hormones in range	L-thyroxine in TSH ≥10 mIU/L subset	Matched euthyroid controls and baseline	Follow-up after treatment in subset	Lipids, glycemia, HOMA-IR, hepatic indices, SCORE2 and lifetime cardiovascular risk, biomarkers
Chen et al. [44]	United States/China; NHANES cross-sectional analysis with animal experiment	NHANES adults n = 3,135; SCH n = 104	TSH >4.5 mIU/L with FT4 within reference range	AIP quartile/exposure; human component only	Lowest AIP quartile	Not applicable	AIP, SCH prevalence, thyroid hormones, lipid measures
Janovsky et al. [45]	Brazil; cross-sectional ELSA-Brasil analysis	Adults n = 3,979; SCH n = 377	TSH >4.0 mIU/L with FT4 within reference range	SCH status and thyroid-related parameters	Euthyroid participants	Not applicable	Metabolic, malnutrition, and inflammation vulnerability indices, FT3, FT4, FT3/FT4 ratio, TPOAb
Acharya et al. [46]	India; cross-sectional study	Women with PCOS n = 368; SCH n = 52	TSH >4.5 mIU/L with normal FT4	SCH status in PCOS	Euthyroid PCOS	Not applicable	BMI, BP, glucose, HbA1c, insulin, HOMA-IR, lipid and reproductive hormones
Sharaf et al. [47]	United States; retrospective multicenter cohort	Hospitalized adults n = 11,118; SCH n = 1,481	TSH 3.75-9.99 mIU/L with normal FT4	SCH status	Euthyroid hospitalized patients	Index admission and 30-day readmission	MACE, in-hospital mortality, length of stay, 30-day readmission
Zielinski et al. [48]	United States; retrospective cohort	Mild SCH n = 52; treated n = 14, untreated n = 38	TSH 4.5-10.0 mIU/L with normal FT4	Levothyroxine treatment	Untreated SCH	At least two TSH/lipid measurements	Total cholesterol, LDL-C, HDL-C, triglycerides
Jain et al. [49]	India; cross-sectional study	Postmenopausal women n = 142; SCH n = 32	Elevated TSH with normal FT4	SCH status	Euthyroid women	Not applicable	BMI, dyslipidemia, hypertension, menopausal duration
Albulushi et al. [50]	Oman; cross-sectional imaging study	Adults with SCH n = 500	TSH 4.5-10 mIU/L with normal FT4	Clinical and biochemical risk factors within SCH	No euthyroid comparator	Not applicable	CIMT, carotid plaque, coronary artery calcium, LDL-C, hs-CRP, metabolic syndrome
Jameel et al. [51]	Pakistan; observational cohort/cross-sectional assessment	Obese adults n = 200; SCH n = 82	TSH >4.5 mIU/L with normal FT4	SCH status in obesity	Obese adults without SCH	Six-month recruitment period; no longitudinal outcome	BMI, fasting glucose, HOMA-IR, lipid profile
Ao et al. [52]	Europe; post-hoc pooled analysis of two double-blind RCTs	Older adults n = 286; levothyroxine n = 142	Persistent SCH in TRUST/IEMO80+; elevated TSH with normal FT4	Levothyroxine titrated to TSH	Placebo	12 months	ApoB, conventional lipids, remnant cholesterol, nuclear magnetic resonance metabolomics
Mawlood [53]	Iraq; cross-sectional comparative study	SCH n = 20 and euthyroid controls n = 20	Elevated TSH with normal FT3/FT4	SCH status	Euthyroid controls	Not applicable	TC, TG, LDL-C, HDL-C, LDL/HDL ratio
Kottola et al. [54]	India; case-control study	SCH n = 150 and matched controls n = 150	TSH 4.5-10 mIU/L with normal FT4	SCH status	Age- and sex-matched healthy controls	Not applicable	TSHR expression, IL-6, 3-nitrotyrosine, TSH
Iram et al. [55]	Pakistan; cross-sectional observational study	PCOS-SCH n = 35, PCOS-euthyroid n = 35, healthy controls n = 35	Elevated TSH with normal free T4/T3	SCH status in PCOS	PCOS-euthyroid and healthy controls	Not applicable	BMI, glucose, HbA1c, lipids, reproductive hormones, gene expression
Martins et al. [56]	Brazil; retrospective cohort	CABG patients n = 863; SCH n = 59, euthyroid n = 804	Elevated age-specific TSH with normal FT4	SCH status before CABG	Euthyroid CABG patients	Postoperative and longitudinal outcomes	MACE, stroke, AF, pleural/pericardial effusion, infection, mortality
Doğan and Aslan [57]	Turkey; cross-sectional observational study	Adults n = 222; SCH n = 59	Thyroid status based on TSH and FT4	SCH status	Overt hypothyroid, euthyroid, and healthy groups	Not applicable	TyG index, lipids, glucose, CRP/albumin ratio
Abumayyaleh et al. [58]	Germany; retrospective HFmrEF registry	HFmrEF patients n = 1,568; SCH n = 95	TSH >4.5 mU/L with FT4 7.5-23 pmol/L	SCH status	Euthyroid HFmrEF patients	Median 30 months	All-cause mortality, HF-related rehospitalization
Li and Ma [59]	China; retrospective comparative study	T2DM n = 384; T2DM-SCH n = 184, T2DM alone n = 200	Elevated TSH with normal FT4	SCH status in T2DM	T2DM without SCH	Not applicable	HOMA-IR, beta-cell function index, fasting glucose, insulin, TSH
Inayat et al. [60]	Pakistan; cross-sectional study	Adults classified with SCH n = 2,024	TSH >4.0 mIU/L with normal FT4	Metabolic predictors within SCH	No euthyroid comparator	Not applicable	TyG index, HbA1c, lipids, thyroid hormones; predictive modelling
Ghosh et al. [61]	India; comparative biomarker study with nonrandomized treatment follow-up	SCH n = 80, overt hypothyroid n = 59, controls n = 60	TSH >4-10 microIU/mL with FT4 0.8-1.9 ng/dL	Levothyroxine in a SCH subset	Untreated SCH, overt hypothyroid, and healthy controls	6 months for treated subset	IL-1beta, IL-6, ICAM-1, hs-CRP, inflammasome expression
Wang et al. [62]	China; retrospective cross-sectional study	Adults n = 676; SCH n = 78	TSH ≥4.20 mIU/L with normal FT3/FT4	SCH status/TSH strata	Three euthyroid TSH strata	Not applicable	Ox-LDL, homocysteine, lipids, ApoA1, ApoB, Lp(a)
Baran et al. [63]	Poland; prospective observational cohort	Women with normogonadotropic anovulation n = 158	Study-defined SCH: TSH >2.5 mIU/L with normal free thyroid hormones	SCH and thyroid autoimmunity status	Women without SCH within PCOS or hypothalamic-pituitary-ovarian dysfunction groups	Not applicable	CRP, TNF-alpha, interleukins, HOMA-IR, lipids, ovarian indices
Qiu et al. [64]	China; cross-sectional study	Overweight/obese, first-episode drug-naive MDD patients n = 1,026; higher-TSH subgroup n = 140	Study-defined higher-TSH phenotype: TSH >8 mIU/L with normal FT4	Higher-TSH subgroup status	Euthyroid participants with major depressive disorder	Single baseline assessment	Blood pressure, BMI, thyroid hormones, psychiatric measures
Yamada et al. [65]	Japan; cross-sectional reference-range study	More than 8,000 adults assessed using Abbott or Siemens assays	Elevated TSH with normal FT4 under manufacturer or age/sex-specific ranges	SCH classification under alternative reference ranges	Euthyroid status	Not applicable	Dyslipidemia, hypertension, diabetes; classification changes
Nguyen et al. [66]	Vietnam; prospective cohort with reference-range component	Reference cohort n = 754; treated SCH cohort completing follow-up n = 27	Persistent elevated age-specific TSH with normal FT4 on two measurements	Low-dose levothyroxine	Baseline within treated cohort	3 and 6 months	Thyroid-related patient-reported outcomes, TSH, FT4, anthropometric and cardiometabolic parameters

Narrative synthesis

Lipid and Metabolic Outcomes

Dyslipidemia was the most consistently reported cardiometabolic finding across the available evidence. Higher total cholesterol, LDL-C, triglycerides, adverse atherogenic ratios, or dyslipidemia prevalence were reported in several observational populations [27,32,33,36,38,39,43,46,49,51,53,55,62]. Gao et al. identified a higher hypertriglyceridemia risk among women with TSH ≥10 mIU/L [27], while the updated evidence extended these findings to adults with obesity, PCOS, diabetes, and other selected clinical populations [43,46,51,55,59]. However, not all metabolic indices differed by thyroid status: Doğan and Aslan found no significant difference in TyG values across thyroid-function groups despite correlations with triglycerides, LDL-C, and inflammatory markers [57], and the SCH association with metabolic vulnerability in Janovsky et al. did not remain significant after multivariable adjustment [45].

Levothyroxine effects on lipid outcomes were mixed. Selected smaller or nonrandomized studies reported improvement in one or more lipid parameters [19,20,22,29-31,40,43,61], but the changes were often based on within-group comparisons, were modest in absolute magnitude, or occurred in selected high-risk subgroups. For example, Pala et al. reported small changes in total cholesterol from 4.26 to 4.13 mmol/L, LDL-C from 2.03 to 1.97 mmol/L, and HDL-C from 0.83 to 0.85 mmol/L [30]. Placebo-controlled and older-adult evidence did not show a consistent lipid benefit [18,52]. Alzahrani et al. and Zielinski et al. reported increases in total and LDL cholesterol among treated participants [37,48], whereas Nguyen et al. found no meaningful short-term cardiometabolic change [66]. Taken together, the evidence is consistent with improvement in selected lipid parameters in some patients, but not a uniform or substantial benefit across all adults with SCH.

Endothelial Function and Vascular Outcomes

Endothelial and vascular findings varied by outcome and follow-up. Niknam et al. reported an increase in flow-mediated dilation from 4.95 ± 2.02% to 9.06 ± 2.85% after levothyroxine, without short-term change in IMT [17]. Cabral et al. observed stable flow-mediated dilation in the treated group while untreated participants declined, suggesting a potential benefit on endothelial function rather than proof of a protective effect [28]. Orel and Martynyuk also reported improved flow-mediated dilation, although levothyroxine was combined with antihypertensive therapy [22]. Albulushi et al. found imaging-defined subclinical atherosclerosis in 46% of adults with SCH, with LDL-C >160 mg/dL, hs-CRP >3 mg/L, TSH >7 mIU/L, and metabolic syndrome independently associated with imaging abnormalities [50].

Structural and molecular vascular evidence was less consistent. Yasar et al. reported higher carotid IMT and changes in homocysteine and apelin [26], whereas the blinded trial by Blum et al. showed no treatment effect on CIMT or plaque burden in older adults [24]. Chen et al. linked higher AIP with SCH in the human National Health and Nutrition Examination Survey (NHANES) component [44], and Wang et al. observed higher ox-LDL, homocysteine, LDL-C, and ApoB with rising TSH [62]. Kottola et al. reported higher TSHR expression, IL-6, and 3-nitrotyrosine in SCH, although only TSH remained an independent predictor in the multivariable model [54]. These findings support potential vascular and proatherogenic associations but do not establish that correcting TSH reverses structural atherosclerosis.

Cardiac Function and Heart Failure Outcomes

Cardiac findings depended strongly on population and outcome. Evran et al. showed a favorable treatment-related pattern in which selected echocardiographic and lipid measures remained stable in treated participants while controls worsened; however, the design does not establish prevention of deterioration [21]. In contrast, Gencer et al. found no improvement in left ventricular ejection fraction or diastolic function in older adults with mild SCH [25]. Rahm et al. reported atrial fibrillation prevalence of 44.6% among participants with TSH >10 mIU/L compared with 32.1% among those with TSH 4-10 mIU/L and identified elevated TSH as an independent correlate of atrial fibrillation [42]. Sharaf et al. found no statistically significant adjusted association with major adverse cardiovascular events, mortality, length of stay, or 30-day readmission among hospitalized patients [47].

In higher-risk cardiovascular populations, Wang et al. reported greater improvement in six-minute walk distance and New York Heart Association class when low-dose levothyroxine was added to standard therapy for heart failure with reduced ejection fraction [41]. Tan et al. associated SCH with lower flow-mediated dilation and more cardiovascular events in heart failure with preserved ejection fraction [35]. Abumayyaleh et al. associated SCH with higher all-cause mortality in heart failure with mildly reduced ejection fraction (adjusted HR: 1.454; 95% CI: 1.001-2.113), but not with heart-failure-related rehospitalization [58]. Martins et al. reported major adverse cardiovascular events in 20.3% of patients with SCH compared with 8.2% of euthyroid patients after coronary artery bypass grafting, alongside higher frequencies of stroke, atrial fibrillation, effusions, and infections [56]. These results suggest that SCH may identify a higher-risk phenotype in selected cardiac populations, but treatment effectiveness and causality require better-controlled evidence.

Insulin Resistance and Broader Cardiometabolic Risk

Insulin resistance and broader metabolic abnormalities were reported in several selected populations. Kirac et al. and Hewage et al. found higher HOMA-IR and adverse lipid indices in obese or young women with SCH [34,39]. Acharya et al., Jameel et al., and Iram et al. reported higher BMI, glycemic indices, HOMA-IR, or dyslipidemia in PCOS or obese cohorts [46,51,55]. Li and Ma found higher HOMA-IR and altered beta-cell indices in T2DM with SCH [59], while Inayat et al. linked TyG with HbA1c and lipid measures within a large SCH sample, although the cross-sectional design and reporting limitations reduce causal interpretation [60]. Pandrc et al. reported improvement from baseline in several measured outcomes after levothyroxine; this was interpreted as improvement in selected metabolic markers rather than a broad metabolic treatment effect [29]. Qiu et al. associated a study-defined TSH >8 mIU/L phenotype with higher systolic blood pressure in overweight/obese MDD [64], whereas Yamada et al. showed that SCH-metabolic associations varied according to assay and reference-range definition [65].

Inflammatory and Biomarker Outcomes

The updated evidence broadened the inflammatory and biomarker domain. Baran et al. associated study-defined SCH with higher TNF-alpha and CRP in women with anovulation, although the low TSH threshold (>2.5 mIU/L) raises concern for exposure misclassification [63]. Ghosh et al. identified IL-1beta, IL-6, and ICAM-1 as predictors of SCH and reported lower inflammatory markers in a nonrandomized treated subset [61]. Kottola et al. and Wang et al. identified oxidative, inflammatory, and proatherogenic biomarker patterns [54,62]. These results are biologically informative but remain predominantly cross-sectional or based on nonrandomized treatment comparisons.

Risk-of-Bias Assessment

Risk of bias was assessed for the principal cardiometabolic result, or evidence stream emphasized in each report. Randomized evidence was assessed using RoB 2, nonrandomized interventional and before-and-after evidence using ROBINS-I, and observational exposure evidence using the predefined observational quality checklist. Each report was assigned to the framework corresponding to the primary evidence stream synthesized here; secondary components were interpreted according to their design limitations. Judgments from different tools were interpreted within their respective design categories and were not treated as directly comparable numerical rankings. The RoB 2 judgments for randomized reports are presented in Table 5.

**Table 5:** RoB 2 assessment for randomized reports RoB 2: Risk of Bias 2; TRUST: Thyroid Hormone Replacement for Untreated Older Adults With Subclinical Hypothyroidism Risk-of-bias assessment for the principal cardiometabolic result evaluated in each randomized report using the Cochrane Risk of Bias 2 tool. Judgments were categorized as low risk, some concerns, or high risk. Ao et al. was assessed as a secondary pooled report of TRUST and IEMO80+

Study ID	Randomization process	Deviations from intended intervention	Missing outcome data	Outcome measurement	Selection of reported result	Overall judgment	Main concerns
Shahebrahimi et al. [18]	Low risk	Low risk	Some concerns	Low risk	Some concerns	Some concerns	Double-blind placebo-controlled design, but follow-up duration reporting appears inconsistent
Evran et al. [21]	Some concerns	Some concerns	Some concerns	Some concerns	Some concerns	Some concerns	Open-label design, small sample, and unclear allocation concealment/blinding details.
Stott et al. [23]	Low risk	Low risk	Low risk	Low risk	Low risk	Low risk	Large blinded placebo-controlled trial with clear outcome assessment.
Blum et al. [24]	Low risk	Low risk	Low risk	Low risk	Low risk	Low risk	Randomized placebo-controlled substudy with objective imaging endpoints.
Gencer et al. [25]	Low risk	Low risk	Low risk	Low risk	Low risk	Low risk	Randomized placebo-controlled substudy with standardized echocardiographic outcomes.
Cabral et al. [28]	Some concerns	Some concerns	Some concerns	Some concerns	Some concerns	Some concerns	Small open-label randomized trial with no-treatment comparator.
Wang et al. [41]	Some concerns	Some concerns	Some concerns	Some concerns	Some concerns	Some concerns	Multicenter randomized trial, but open-label and without placebo control.
Ao et al. [52]	Low risk	Low risk	Some concerns	Low risk	Some concerns	Some concerns	Post-hoc pooled biomarker analysis; biobank availability, multiple outcomes, and exploratory TSH-stratified analyses.

ROBINS-I judgments for nonrandomized interventional reports are presented in Table 6.

**Table 6:** ROBINS-I assessment for nonrandomized interventional evidence ROBINS-I: Risk of Bias in Nonrandomized Studies of Interventions​​​ Risk-of-bias assessment for the principal treatment-effect evidence from nonrandomized interventional and before-and-after reports using ROBINS-I. Judgments were categorized as low, moderate, serious, or critical risk of bias. The category some concerns was not used because it applies to RoB 2

Study ID	Confounding	Participant selection	Intervention classification	Deviations from intervention	Missing data	Outcome measurement	Reported result selection	Overall judgment	Main concerns
Niknam et al. [17]	Moderate	Moderate	Low	Moderate	Moderate	Low	Moderate	Moderate	Quasi-experimental design, small sample, and short follow-up
Beysel et al. [19]	Moderate	Moderate	Low	Moderate	Moderate	Low	Moderate	Moderate	Retrospective before-after design and possible confounding
Adamarczuk-Janczyszyn et al. [20]	Moderate	Moderate	Low	Moderate	Moderate	Low	Moderate	Moderate	Nonrandomized before-after design in obese postmenopausal women
Orel and Martynyuk [22]	Serious	Moderate	Low	Moderate	Moderate	Low	Moderate	Serious	No control group and combined levothyroxine with antihypertensive therapy
Yasar et al. [26]	Moderate	Moderate	Low	Moderate	Moderate	Low	Moderate	Moderate	Nonrandomized reassessment after levothyroxine with surrogate biomarkers
Pandrc et al. [29]	Serious	Moderate	Low	Moderate	Moderate	Low	Moderate	Serious	Small pilot before-after study without separate control group
Pala et al. [30]	Moderate	Moderate	Low	Moderate	Moderate	Low	Moderate	Moderate	Treatment limited to TSH >10 group and nonrandomized allocation
Maurya et al. [31]	Serious	Moderate	Low	Moderate	Moderate	Low	Moderate	Serious	Small treated SCH group; healthy controls did not provide randomized treatment allocation, and treatment effects were based mainly on pre-post comparisons
Alzahrani et al. [37]	Moderate	Moderate	Low	Moderate	Moderate	Low	Moderate	Moderate	Retrospective treatment analysis and unclear treatment follow-up timing
Serdar Hiršl et al. [40]	Moderate	Moderate	Low	Moderate	Moderate	Low	Moderate	Moderate	Nonrandomized treatment decision and small treated subgroup.
Tsilingiris et al. [43]	Serious	Moderate	Low	Moderate	Moderate	Low	Moderate	Serious	Nonrandomized treatment of a small TSH ≥10 subgroup; confounding by indication and limited power
Zielinski et al. [48]	Serious	Moderate	Moderate	Moderate	Moderate	Low	Moderate	Serious	Retrospective treated-versus-untreated comparison with confounding by indication and small treated group
Ghosh et al. [61]	Serious	Moderate	Low	Moderate	Moderate	Low	Moderate	Serious	Nonrandomized treated and untreated SCH subsets; incomplete subgroup-size and cointervention detail
Nguyen et al. [66]	Serious	Moderate	Low	Moderate	Moderate	Low	Moderate	Serious	Single-group before-after cohort with 27 completers and no concurrent control

Observational quality assessment judgments are presented in Table 7.

**Table 7:** Observational quality assessment judgments RoB 2: Risk of Bias 2; ROBINS-I: Risk of Bias in Nonrandomized Studies of Interventions Domain-level judgments for the principal observational exposure evidence. Ratings were interpreted within the observational framework and are not directly interchangeable with RoB 2 or ROBINS-I categories

Study ID	Selection/sampling	Comparability and confounding	Exposure definition	Outcome measurement	Statistical analysis	Overall judgment
Gao et al. [27]	Some concerns	Moderate concerns	Low concern	Low concern	Some concerns	Moderate concerns
Goyal et al. [32]	Moderate concerns	Moderate concerns	Low concern	Low concern	Some concerns	Moderate concerns
Ejaz et al. [33]	Moderate concerns	Moderate concerns	Low concern	Low concern	Some concerns	Moderate concerns
Kirac et al. [34]	Some concerns	Moderate concerns	Low concern	Low concern	Some concerns	Some concerns
Tan et al. [35]	Some concerns	Moderate concerns	Low concern	Low concern	Low concern	Moderate concerns
Harrar et al. [36]	Moderate concerns	Moderate concerns	Low concern	Low concern	Some concerns	Moderate concerns
Zhu and Jiang [38]	Some concerns	Moderate concerns	Low concern	Low concern	Some concerns	Moderate concerns
Hewage et al. [39]	Moderate concerns	Moderate concerns	Low concern	Low concern	Some concerns	Moderate concerns
Rahm et al. [42]	Some concerns	Moderate concerns	Low concern	Low concern	Some concerns	Moderate concerns
Chen et al. [44]	Some concerns	Some concerns	Low concern	Low concern	Some concerns	Some concerns
Janovsky et al. [45]	Some concerns	Low concern	Low concern	Low concern	Low concern	Some concerns
Acharya et al. [46]	Moderate concerns	Moderate concerns	Low concern	Low concern	Some concerns	Moderate concerns
Sharaf et al. [47]	Some concerns	Moderate concerns	Low concern	Low concern	Low concern	Moderate concerns
Jain et al. [49]	Moderate concerns	Moderate concerns	Low concern	Low concern	Some concerns	Moderate concerns
Albulushi et al. [50]	Some concerns	Moderate concerns	Low concern	Low concern	Low concern	Moderate concerns
Jameel et al. [51]	Moderate concerns	Moderate concerns	Low concern	Low concern	Some concerns	Moderate concerns
Mawlood [53]	Moderate concerns	Moderate concerns	Low concern	Low concern	Some concerns	Moderate concerns
Kottola et al. [54]	Moderate concerns	Moderate concerns	Low concern	Low concern	Some concerns	Moderate concerns
Iram et al. [55]	Moderate concerns	Moderate concerns	Low concern	Low concern	Some concerns	Moderate concerns
Martins et al. [56]	Some concerns	Moderate concerns	Low concern	Low concern	Some concerns	Moderate concerns
Doğan and Aslan [57]	Moderate concerns	Moderate concerns	Low concern	Low concern	Some concerns	Moderate concerns
Abumayyaleh et al. [58]	Some concerns	Moderate concerns	Low concern	Low concern	Low concern	Moderate concerns
Li and Ma [59]	Moderate concerns	Moderate concerns	Low concern	Low concern	Some concerns	Moderate concerns
Inayat et al. [60]	Serious concerns	Serious concerns	Some concerns	Low concern	Serious concerns	Serious concerns
Wang et al. [62]	Some concerns	Moderate concerns	Low concern	Low concern	Some concerns	Moderate concerns
Baran et al. [63]	Moderate concerns	Moderate concerns	Serious concerns	Low concern	Some concerns	Serious concerns
Qiu et al. [64]	Moderate concerns	Moderate concerns	Serious concerns	Low concern	Some concerns	Serious concerns
Yamada et al. [65]	Some concerns	Some concerns	Some concerns	Low concern	Low concern	Some concerns

The principal methodological concerns for observational reports are summarized in Table 8.

**Table 8:** Main methodological concerns in observational reports SCH: subclinical hypothyroidism Main methodological concerns accompanying the domain-level judgments in Table 7A. Mixed human/experimental reports were assessed only for their eligible human component

Study ID	Main methodological concerns
Gao et al. [27]	Cross-sectional design, women-only sample, and limited confounding control
Goyal et al. [32]	Small SCH subgroup and retrospective cross-sectional design
Ejaz et al. [33]	Single-hospital study and nonprobability sampling
Kirac et al. [34]	Small retrospective study restricted to obese women
Tan et al. [35]	Prospective cohort with clinical outcomes, but residual confounding remains possible
Harrar et al. [36]	Retrospective hospital-record design and broad clinical population
Zhu and Jiang [38]	Retrospective community study with possible residual confounding
Hewage et al. [39]	Cross-sectional design and self-selected young female population
Rahm et al. [42]	Retrospective clinical cohort; AF prevalence and residual confounding; experimental component interpreted separately
Chen et al. [44]	NHANES cross-sectional analysis; temporality unavailable; human component only
Janovsky et al. [45]	Large cohort with adjustment, but cross-sectional and multiple-comparison sensitivity reduced robustness
Acharya et al. [46]	Single-center cross-sectional PCOS cohort with residual confounding
Sharaf et al. [47]	Large retrospective hospitalized cohort; residual confounding and single-admission classification
Jain et al. [49]	Small cross-sectional postmenopausal cohort with limited adjustment
Albulushi et al. [50]	Cross-sectional SCH-only imaging cohort without euthyroid comparator; association rather than prevalence comparison
Jameel et al. [51]	Single-center obese cohort; cross-sectional metabolic assessment despite prospective recruitment terminology
Mawlood [53]	Very small convenience sample and limited confounder adjustment
Kottola et al. [54]	Case-control biomarker study with uncertain external validity and residual confounding
Iram et al. [55]	Small cross-sectional groups; PCOS itself complicates attribution of metabolic differences to SCH
Martins et al. [56]	Retrospective CABG cohort; residual confounding and unadjusted event-frequency interpretation
Doğan and Aslan [57]	Cross-sectional thyroid-state comparison; modest subgroup sizes and no temporal inference
Abumayyaleh et al. [58]	Large retrospective HF registry with multivariable adjustment but possible residual confounding
Li and Ma [59]	Retrospective T2DM comparison; diagnostic-model analyses do not establish directionality
Inayat et al. [60]	Convenience sample, internal wording inconsistencies, no euthyroid comparator, and data/model reporting concerns
Wang et al. [62]	Retrospective cross-sectional biomarker study with unequal groups and residual confounding
Baran et al. [63]	Nonstandard TSH >2.5 mIU/L SCH threshold creates substantial exposure-misclassification concern
Qiu et al. [64]	Single baseline measurement and exploratory TSH >8 mIU/L phenotype not equivalent to guideline-confirmed SCH
Yamada et al. [65]	Large reference-range analysis; associations were assay- and classification-dependent

Within the randomized evidence, the TRUST reports received low-risk RoB 2 judgments [23-25], whereas the pooled biomarker report raised some concerns because of biobank availability, multiple outcomes, and exploratory TSH-stratified analyses [52]. Judgments were interpreted within their design categories rather than as direct rankings across tools. Smaller randomized or open-label studies raised concerns about sample size, blinding, allocation reporting, or comparators. Nonrandomized treatment studies often had serious risk of bias because of confounding and the absence of concurrent controls. Observational studies supported associations but could not establish temporality or causality; key concerns were nonstandard SCH definitions, convenience samples, and incomplete control of confounding.

Discussion

Principal Findings

This systematic review included 50 reports covering 48 distinct study populations and at least 46,746 participants. The evidence comprised eight randomized reports, 14 nonrandomized interventional reports, and 28 observational reports. Across the included populations, SCH was associated with adverse lipid, vascular, metabolic, and cardiovascular findings, but the direction and magnitude of these associations varied according to the population, outcome, biochemical definition, and study design. Dyslipidemia was the most consistently reported finding. Selected treatment studies reported improvements in lipid levels, endothelial function, blood pressure, inflammatory biomarkers, or functional outcomes, although many relied on small samples, within-group comparisons, or nonrandomized treatment allocation. In contrast, large placebo-controlled studies in older adults with mild SCH found no meaningful improvement in symptoms, carotid atherosclerosis, cardiac function, or overall cardiometabolic biomarkers despite biochemical reductions in TSH [23-25,52].

The updated evidence therefore supports a heterogeneous and subgroup-dependent cardiometabolic phenotype rather than a uniformly treatment-responsive condition. Larger event differences and adverse cardiometabolic profiles were reported in selected populations with obesity, diabetes, heart failure, or cardiac surgery, but residual confounding limits causal interpretation [35,46,47,50,51,55-60]. Treatment effects also varied according to age, persistence and degree of TSH elevation, baseline cardiovascular risk, comorbidities, and the outcome assessed. These findings favor individualized clinical assessment rather than routine treatment aimed solely at normalizing TSH.

Comparison With Previous Reviews and Guidelines

The findings are broadly consistent with the Cochrane review of thyroid hormone replacement for SCH, which did not demonstrate improved survival, cardiovascular morbidity, symptoms, or quality of life, although possible improvements were noted in selected lipid and cardiac measures [12]. The present review extends that earlier evidence by incorporating larger blinded trials, contemporary biomarker analyses, and studies in metabolically and cardiovascularly high-risk populations. The newer randomized evidence strengthens the conclusion that biochemical TSH reduction does not necessarily translate into broad symptomatic, vascular, or cardiac benefit in older adults with mild SCH [23-25,52].

The results also align with the risk-stratified approach adopted by major clinical guidance. The American Association of Clinical Endocrinologists and American Thyroid Association guideline recommends that treatment decisions for patients with TSH below 10 mIU/L be individualized [4]. The European Thyroid Association guideline distinguishes mild TSH elevation from values above 10 mIU/L, supports treatment more strongly in younger patients with persistent TSH above 10 mIU/L, and emphasizes age-specific reference ranges and a cautious approach in the oldest adults [5]. The present synthesis supports these distinctions because adverse associations and measurable biochemical treatment responses were more evident in some higher-TSH or higher-risk groups, whereas the strongest evidence in older adults with mild SCH did not support routine treatment.

Dyslipidemia and Broader Metabolic Risk

Dyslipidemia was reported across diverse populations, including community samples, hospital cohorts, women with PCOS or menopause-related characteristics, and adults with obesity or diabetes [27,32,33,36,38,39,43,46,49,51,53,55,59,62]. Higher total cholesterol, LDL-C, triglycerides, adverse atherogenic ratios, or a greater prevalence of dyslipidemia were reported in several studies. The relationship appeared partly severity-dependent, with more pronounced lipid abnormalities or hypertriglyceridemia reported in groups with TSH concentrations of 10 mIU/L or higher [27,30]. However, these findings were not universal, and some adjusted associations weakened or disappeared after accounting for other metabolic factors [45,57].

The metabolic findings similarly varied by population. Higher insulin resistance indices, glycemic measures, BMI, or adverse lipid profiles were reported in selected cohorts with obesity, PCOS, and type 2 diabetes [34,39,46,51,55,59,60]. These associations may reflect an interaction between thyroid status and pre-existing metabolic vulnerability rather than an effect of SCH alone. Differences in age, adiposity, diabetes status, medication use, and baseline cardiovascular risk likely contributed to the variation across studies.

Levothyroxine Treatment and Potential Effect Modifiers

Levothyroxine treatment did not produce a uniform cardiometabolic response. Several smaller or nonrandomized studies reported improvements in selected lipid parameters, blood pressure, endothelial function, inflammatory markers, or other measured outcomes [19,20,22,29-31,40,43,61]. These changes were often based on within-group comparisons, were modest in absolute magnitude, or occurred in selected subgroups. Conversely, two retrospective treatment studies reported increases in total and LDL-C after treatment [37,48], and placebo-controlled or pooled older-adult evidence did not show a consistent lipid or broader biomarker benefit [18,52]. These discordant findings show that statistical significance should be interpreted alongside effect size, study design, baseline risk, and clinical relevance.

Vascular and cardiac findings showed a similar pattern. Small studies suggested a potential benefit on flow-mediated dilation [17,22,28], but the blinded trial in older adults did not show improvement in CIMT or plaque burden [24]. In heart failure with reduced ejection fraction, adding low-dose levothyroxine to standard therapy improved exercise capacity and New York Heart Association class in one open-label randomized study [41]. This contrasts with the absence of improvement in resting cardiac function among older adults with mild SCH [25]. The apparent benefit in selected high-risk populations therefore requires confirmation in adequately powered blinded trials with clinically meaningful endpoints.

Age and biochemical severity were important sources of variation. Older adults with mild SCH were less likely to benefit from routine treatment in the largest randomized studies [23-25,52]. In contrast, some studies reported stronger adverse lipid associations or greater biochemical changes when TSH was 10 mIU/L or higher [27,30,43]. Nevertheless, the included literature used heterogeneous diagnostic thresholds, assay-specific reference ranges, and both free and total thyroid hormone measurements. Some studies confirmed persistent abnormalities on repeated testing, whereas others relied on a single assessment. These differences limit direct comparison and reinforce the need to interpret TSH in relation to age, persistence, assay, thyroid hormone concentration, and clinical context.

Clinical Implications

The findings support a risk-based approach to SCH rather than a uniform treatment strategy. TSH should be interpreted alongside age, persistence of elevation, symptoms, thyroid autoimmunity, lipid profile, cardiovascular risk, heart failure status, blood pressure, obesity, insulin resistance, and patient preferences. Treatment should not be based solely on biochemical normalization, particularly in older adults with mild SCH, for whom randomized evidence has not shown meaningful symptomatic or cardiovascular surrogate benefit [23-25,52].

Levothyroxine may be considered as part of individualized management in younger patients, those with persistent TSH concentrations of 10 mIU/L or higher, and selected patients with dyslipidemia, endothelial dysfunction, hypertension, or established cardiac disease. However, much of the apparent benefit in these subgroups comes from small, open-label, nonrandomized, or selected-population studies. Thyroid treatment should therefore complement rather than replace standard cardiometabolic risk management, including lipid control, blood pressure management, weight management, glucose assessment, and optimization of cardiovascular therapy.

Limitations

This review has several limitations. The included studies were clinically and methodologically heterogeneous in design, participant characteristics, biochemical definitions, comparator groups, treatment duration, follow-up, outcome definitions, and measurement units. SCH thresholds ranged from relatively low study-defined cutoffs to values above 10 mIU/L, and thyroid status was established using different assays and combinations of FT4, total T4, or total T3 and T4. This diagnostic heterogeneity, together with inconsistent confirmation of persistent TSH elevation, may have introduced exposure misclassification and limited direct comparison. It also prevented meaningful meta-analysis.

Many positive treatment findings came from small, open-label, nonrandomized, or before-and-after studies. These designs are vulnerable to confounding by indication, baseline imbalance, regression to the mean, cointerventions, selection bias, and selective outcome reporting. Important confounders were not consistently measured or adjusted, including baseline BMI, hepatic steatosis or liver function, renal function, lipid-lowering and cardiometabolic medications, thyroid autoimmunity, diabetes, and established cardiovascular disease. Autoimmune status may influence both persistence of SCH and cardiometabolic risk, but it was reported inconsistently.

Several studies relied on single-time-point thyroid, inflammatory, vascular, or metabolic biomarkers. Such measurements may reflect transient biological variation, intercurrent illness, or assay variability and may not represent persistent disease. In addition, many studies assessed surrogate outcomes, including lipid levels, flow-mediated dilation, CIMT, inflammatory markers, insulin resistance indices, and echocardiographic parameters, rather than myocardial infarction, stroke, cardiovascular mortality, or all-cause mortality. Changes in surrogate markers do not necessarily translate into long-term clinical benefit.

Publication bias could not be formally assessed because meta-analysis was not undertaken and comparable effect estimates were insufficient for funnel-plot or regression-based methods. Positive results from small or uncontrolled studies may therefore be overrepresented. The review was restricted to English-language reports, Google Scholar screening was limited to the first 100 relevance-ranked results, and the update search used PubMed/MEDLINE with supplementary Scopus discovery rather than rerunning every original database. Seven potentially relevant reports identified during the update could not be retrieved despite reasonable attempts. Finally, formal certainty-of-evidence grading was not performed, so interpretation relied on study design, risk of bias, consistency, directness, sample size, and clinical relevance.

Future Research

Future research should prioritize adequately powered blinded randomized trials in younger adults, patients with persistent TSH concentrations of 10 mIU/L or higher, and patients with clearly defined cardiometabolic or heart failure phenotypes. Studies should use repeated biochemical confirmation, assay-appropriate and age-specific reference ranges, standardized outcome definitions, longer follow-up, and clinically meaningful cardiovascular endpoints. Thyroid autoimmunity should be measured consistently, and analyses should prespecify adjustment for adiposity, diabetes, hepatic and renal function, lipid-lowering therapy, and established cardiovascular disease. Greater attention to effect modification by age, sex, baseline TSH, autoimmunity, obesity, diabetes risk, lipid profile, and cardiac phenotype would help identify patients most likely to benefit from treatment.

## Conclusions

SCH has been associated with adverse cardiometabolic findings, particularly dyslipidemia, endothelial dysfunction, insulin resistance, and vascular biomarker abnormalities. Associations with cardiovascular risk were more evident in selected higher-risk populations and varied according to age, baseline TSH level, comorbidities, SCH definition, and study design. The strongest available evidence from lower-risk randomized trials in older adults with mild SCH showed no clinically meaningful benefit of levothyroxine for symptoms, carotid atherosclerosis, cardiac function, or broader cardiometabolic outcomes despite biochemical reductions in TSH. Benefits reported in younger patients, those with more marked TSH elevation, or patients with specific cardiometabolic comorbidities were derived mainly from smaller or nonrandomized studies and should therefore be considered hypothesis-generating rather than established. The findings support individualized, risk-based management rather than routine treatment based on TSH elevation alone.
